# Does previous total hip arthroplasty affect the outcomes of total knee arthroplasty? A one- and five-year follow-up registry study in a monocentric hospital

**DOI:** 10.1186/s13018-024-04923-5

**Published:** 2024-07-27

**Authors:** Leena Ristolainen, Jyrki Kettunen, Jouni Lohikoski, Hannu Kautiainen, Mikko Manninen

**Affiliations:** 1 Orton Orthopaedic Hospital, Tenholantie 10, Helsinki, 00280 Finland; 2 Arcada University of Applied Sciences, Jan-Magnus Janssonin aukio 1, Helsinki, 00550 Finland; 3 Primary Health Care Unit, Kuopio University Hospital, P.O. Box 100, Kuopio, 70029 KYS FI Finland; 4 Folkhälsan Research Center, Topeliuksenkatu 20, Helsinki, 00250 Finland

**Keywords:** Total knee arthroplasty, Total hip arthroplasty, Pain, Physical function, Walking distance, Hungerford score

## Abstract

**Background:**

Osteoarthritis in the lower extremities becomes more common as people age. In addition to conservative treatments, hip or knee arthroplasty is often needed. The aim of this study was to evaluate total knee arthroplasty (later TKA) in patients, comparing those who had previously undergone THA (later THA/TKA), with those who had not undergone such procedure. Pain, walking ability and functional capacity were assessed.

**Methods:**

Patients who underwent primary TKA between 1987 and 2017 at a single orthopaedic hospital was included in this study. The patients participated in clinical preoperative and postoperative examinations by an orthopaedic surgeon after one- and five- years. The final study group consisted of 418 patients who had undergone 502 knee arthroplasties. Of these 502 TKA cases, 462 had not undergone previous THA and 40 had undergone previous THA. To evaluate the patients’ physical function and walking ability, a structure form for knee arthroplasty based on the Hungerford score was used. The registry data from the Finnish National Institute of Health and Welfare was used. The data included TKA revision(s) and mortality events.

**Results:**

At the baseline and after one- and five- years primary TKA, no statistical differences were found in the total Hungerford score between TKA patients and THA/TKA patients. In both groups, the total score increased per surgery year. However, when analysing the relationship between the year of operation and the total score, no statistical differences were found between the groups (TKA and THA/TKA) at five years (*p* = 0.61). The only statistical difference found between the groups was in walking distance points after one year; THA/TKA patients (mean 83 [SD 17]) could walk remarkably shorter distances than TKA patients (91 [14]) one year after arthroplasty (*p* < 0.001).

**Conclusions:**

In conclusion, walking distance improved more rapidly in TKA patients than in THA/TKA patients. However, patients who underwent more than one arthroplasty in their lower extremities managed their lives, activities, and pain almost as well as those who underwent only one knee arthroplasty.

## Background

A meta-analysis by Gross et al. [1] estimated the global burden of hip and knee osteoarthritis (OA) and concluded that factors, such as ageing and obesity, will increase the need for treatment of hip and knee OA. Hip or knee arthroplasty is reported to be an effective treatment for patients with severe symptoms [2]. It has also been reported that the progression from OA to arthroplasty is strongly influenced by factors related to the severity of OA symptoms and the patient’s desire for surgery [3]. Partly due to these factors, some patients may need more than one arthroplasty of the lower extremities.

Jungmann et al. [4] investigated the association of prevalent unilateral total hip arthroplasty (THA) with radiological and functional changes in the knees at baseline (time of initial THA) and during a four-year follow-up. They concluded that prevalent unilateral THA increases the risk of developing OA in the knee contralateral to the THA.

Only a few studies have reported on the clinical outcomes of total knee arthroplasty (TKA) in patients who underwent previous THA [5, 6]. Lee et al. [6] compared the leg length discrepancy (LLD) in TKA patients with or without previous ipsilateral THA. They reported that the LLD was greater in TKA patients who had previously undergone THA than in patients who underwent TKA only, both before TKA and four years after TKA. Asensio-Pascual et al. [5] concluded that a well-functioning unilateral THA does not influence the functional outcome of a subsequent ipsilateral TKA. In a study by Lee et al. [6], 11% of patients who underwent primary TKA had a previous history of ipsilateral THA after a hip fracture. The knowledge of the role of previous THA in recovery after TKA remains limited. Therefore, it was conducted a one- and five-year follow-up study to investigate symptoms and physical function after primary TKA among patients with and without previous THA.

## Materials and methods

### Study design

This study was a retrospective comparative registry-based study in a single orthopaedic hospital.

### Methods

Patients who underwent TKA between 1987 and 2017 at a single orthopaedic hospital in Helsinki, Finland were recruited. As a part of the hospital’s quality control, a voluntary postoperative follow-up examination was available to patients after one- and five- years of TKA.

Overall, 6650 TKAs were conducted during that time. The inclusion criterion were patients who underwent primary TKA due to degenerative OA and completed their one- and five-year follow-up evaluations. In this study, patients without previous THA (later TKA) and patients with previous THA (later THA/TKA) were included. The patients underwent examination by an orthopaedic surgeon before their TKA operations. Then, the patients underwent examination by an orthopaedic surgeon one- and five years after the operation. The exclusion criterion included TKA for other reason than primary OA, also revisions were excluded. Patients who did not have preoperative and one- and five-year postoperative orthopaedic surgeon examinations were also excluded.

The final study group consisted of 418 patients who underwent 502 knee arthroplasties, 462 had not undergone previous THA and 40 had undergone previous THA. 40% of previous THA (16/40) had bilateral hip arthroplasties; 30% had THA on the same side as the TKA, and 30% on the opposite side. The baseline characteristics are shown in Table 1.

**Table 1:** Patient characteristics and sub-items of the Hungerford score at baseline

Variable	TKA* N = 462	THA/TKA** N = 40	*p*-value
Subjects, n	380	38	…
Female, n (%)	325 (70)	23 (58)	0.091
Age, mean (SD)	68 (8)	67 (7)	0.70
BMI, kg/m^2^, mean (SD)	28.5 (4.3)	28.1 (4.1)	0.71
Total Hungerford score, mean (SD)	70.6 (17.5)	66.0 (17.9)	0.11
**Subscales (1–6)**			
*1. Pain*, n (%)			0.39
Frequent pain	56 (12)	5 (13)	
Occasional rest pain	41 (9)	4 (10)	
Pain restricts walking	52 (11)	10 (25)	
Pain in walking	259 (56)	15 (38)	
Slight pain with routine activities	52 (11)	4 (10)	
No pain	2 (0)	2 (5)	
*2. Stability*, n (%)			0.16
> 15°	14 (3)	3 (8)	
5°-15°	83 (18)	9 (23)	
0°-5°	365 (79)	28 (70)	
*3. Deformity*, n (%)			0.56
> 20°	6 (1)	0 (0)	
16°-20°	1 (0)	0 (0)	
11°-15°	101 (22)	12 (30)	
6°-10°	196 (42)	15 (38)	
0°-5°	158 (34)	13 (33)	
*4. Flexion contracture*, n (%)			0.21
> 45°	2 (0)	0 (0)	
30°-45°	5 (1)	0 (0)	
15°-30°	35 (8)	5 (13)	
5°-15°	177 (38)	18 (45)	
0	243 (53)	17 (43)	
*5. Mobility*, n (%)			0.58
1°-30°	1 (0)	1 (3)	
31°-60°	6 (1)	0 (0)	
61°-90°	24 (5)	2 (5)	
90°-105°	35 (8)	4 (10)	
> 105°	396 (86)	33 (83)	
*6. Quadriceps strength*, n (%)			0.28
50%	8 (2)	4 (10)	
50-75%	115 (25)	9 (23)	
> 75%	339 (73)	27 (68)	
*Walking distance*, n (%)			0.34
Unable to walk	2 (0)	0 (0)	
Only at home	20 (4)	2 (5)	
Less than 200	123 (27)	14 (35)	
200–2000 m	294 (64)	22 (55)	
Unlimited	23 (5)	2 (5)	

^*TKA: Total knee arthroplasty; patients had not undergone previous total hip arthroplasty (THA)^

^**THA/TKA: Patients had undergone THA before TKA^

Patient pain and physical function data were systematically collected with the same form during the clinical examinations. The structured form used for knee arthroplasty was based on the Hungerford score [7]. To determine this score, an orthopaedic surgeon examined knee joint stability (measured with maximal extension as the arc of *either* abnormal medical or lateral motion), deformity (varus/valgus deformity), flexion contracture, mobility (the measure of the total passive arc of motion), and quadricep strength (rated subjectively as the percentage of the other side’s quadricep strength). Knee pain was categorised into six classes, with patient-reported pain using a score ranging from 0 (frequent pain) to 50 (no pain at all). The worst knee condition was − 25 points, and the best condition was 100 points. In addition, details regarding patient-reported walking distance, scored from 0 (unable to walk) to 100 (unlimited walking distance) was gathered.

The registry data from the Finnish National Institute of Health and Welfare was used. The data included TKA revision(s) (date and cause) and mortality events [8]. Patients were tracked until the end of the follow-up period, revision, or death.

### Statistical analysis

Summary statistics are presented as the means and standard deviations, medians and interquartile ranges (IQRs), or numbers as percentages. Statistical evaluation of the groups (TKA and THA/TKA) was conducted using Student’s t-test and Pearson’s chi-squared test. Repeated measures of the changes in Hungerford scores were compared between the groups using mixed-effects models and an unstructured covariance structure (i.e., the Kenward–Roger method for calculating the degrees of freedom). Fixed effects included group, time, and group×time interactions. The repeated measurements were taken at different time points, including at baseline, one year, and five years. Mixed models allowed for analysing unbalanced datasets without imputation; therefore, all available data were analysed using the full set of analyses. The relationships between the year of operation and the total score were modelled using linear regression analysis, and the relationships between groups (TKA and THA/TKA) were tested using evaluation the seemingly unrelated estimation (Suest) method. The revision rate functions between the groups were compared using the log-rank test. Revision rates are presented as percentages and 95% confidence intervals (95% CIs). Normally distributed data were evaluated graphically and with the Shapiro–Wilk Wtest. All analyses were performed using Stata 18.0 (StataCorp LP; College Station, TX, USA).

## Results

The final study group consisted of 462 TKAs (380 patients) and 40 THAs/TKAs (38 patients) (Table 1). No group differences were found regarding mean age and gender at baseline (Table 1). Previous hip arthroplasty was performed a median of 2.2 years before (IQR: 4.3–1.2) the primary TKA. At baseline, the total Hungerford score did not differ between the TKA patients and THA/TKA patients (*p* = 0.11). Furthermore, no statistical differences were found in the Hungerford subscale scores between the groups at baseline (Table 1). In both patient groups, the most common symptom was pain when walking. The highest proportion of patients in both groups reported that they could walk 200–2000 m. Only a few patients reported an unlimited walking distance.

**Fig. 1:**
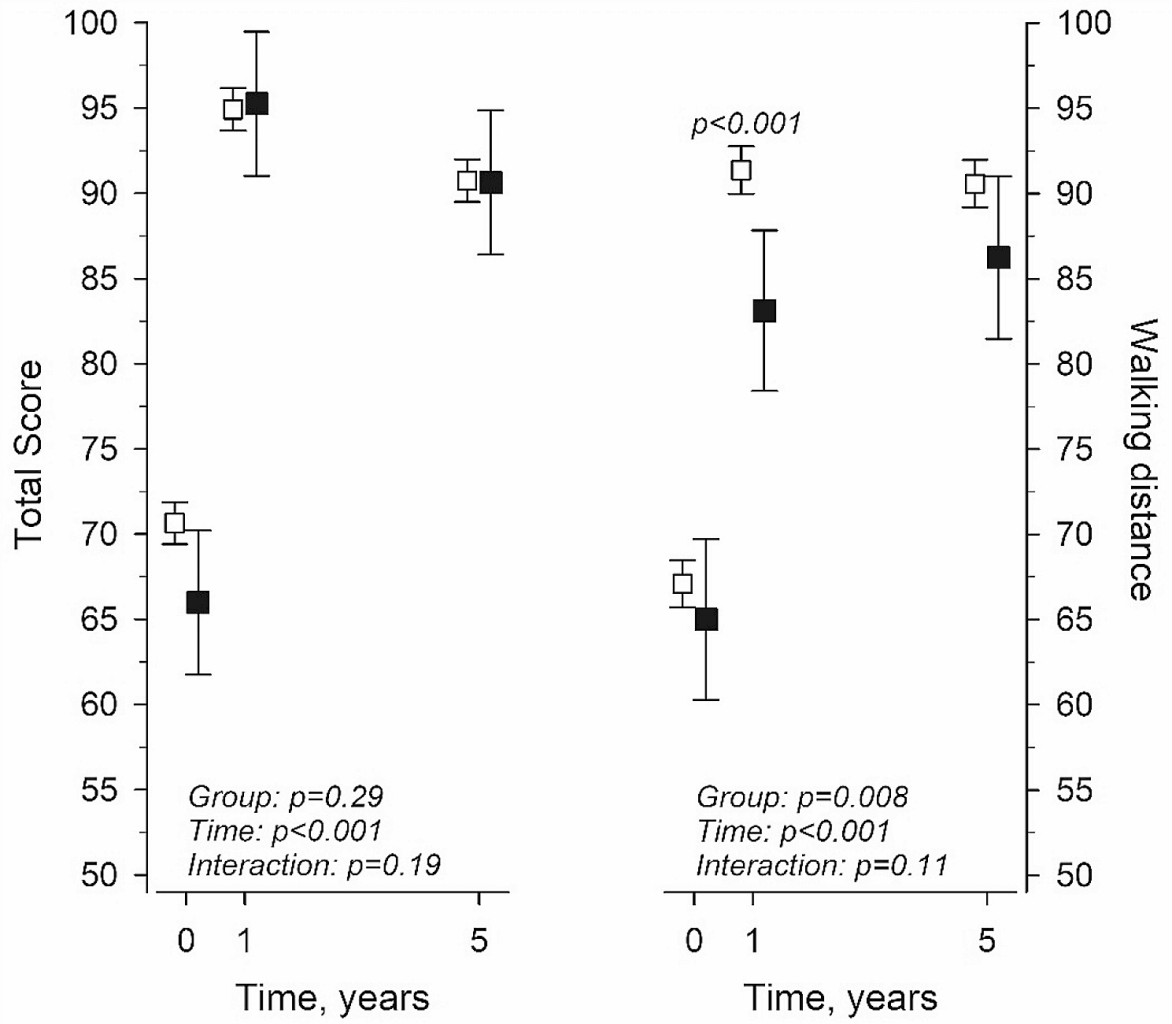
Preoperative, one- and five-year postoperative data for total Hungerford score and walking distance points between TKA patients (white square in the figure) and THA/TKA patients (black square in the figure)

At the one-year follow-up, the mean total Hungerford score increased by 24 points (95% CI: 23–26) in the TKA group and by 29 points (95% CI: 24–35) in the THA/TKA group (*p* = 0.10). The change in total score from baseline to five years was 20 points (95% CI: 18–22) in TKA patients and 25 points (95% CI: 20–30) in THA/TKA patients (*p* = 0.14) (Fig. 1). The total score increased significantly per surgery year in TKA operations (β = 0.27, [95% CI: 0.13–0.40]). Additionally, the total score increased in THA/TKA operations per surgery year, but no statistical differences were found between TKA and THA/TKA groups (β = 0.41 [-0.14–0.96]). However, when comparing total scores between groups (TKA and THA/TKA) at five years, no statistical differences were found (*p* = 0.61).

The only statistical difference between the groups was in walking distance points after one year. THA/TKA patients (mean: 80/100) could walk remarkably shorter distances than TKA patients (91/100) (*p* < 0.001). After five years, THA/TKA patients still had a slightly lower walking ability than TKA patients (Fig. 1).

No statistical difference was found in the change in pain at the five-year follow-up between TKA patients and THA/TKA patients (14 [95% CI: 16–19] vs. 15 [95% CI: 10–20], respectively, *p* = 0.51). The change in walking distance five years after TKA did not differ between the groups; TKA patients had an increase of 24 points (95% CI: 22–25), while THA/TKA patients had an increase of 21 points (95% CI: 16–27) (*p* = 0.45).

After five years, the revision rate was 2% in TKA patients (95% CI: 1–4) and 5% in THA/TKA patients (95% CI: 1–18) (*p* = 0.26). Although, it is not statistically different, it is clinically significant in joint surgery.

## Discussion

The outcomes of pain, knee joint function, and walking distance was investigated in two patient groups: TKA patients who previously underwent THA and patients who underwent only TKA without earlier THA. One year after primary TKA, pain decreased, and physical function and walking distances increased in both groups. The only statistical difference was found in walking distance points after one year, THA/TKA patients walked significantly shorter distances than TKA patients. Five years after TKA, the walking distance of patients who underwent THA/TKA increased, reaching nearly the same level as that of patients in the TKA group. Five years after primary TKA, pain and physical function of the knee joint worsened slightly in both groups. The differences observed did not reach statistical significance.

Both groups (TKA and THA/TKA) had relatively the same number of TKAs per year. and the total score increased per surgery year in both groups. However, this difference did not reach statistical significance.

In this study, the walking distance at the one-year follow-up was significantly worse in the THA/TKA patients than in the TKA patients. Thus, TKA patients improved their walking distance more rapidly than THA/TKA patients. Walking and physical function may be better if only one joint is painful and there are no OA in other lower extremity joints.

Comparing this study with existing literature is challenging, as only few studies have analysed TKA and prior THA [5, 6]. However, other studies have analysed TKA following ipsilateral THA [4, 9]. Due to the small sample size, further analyses to compare more specifically patients with earlier THA and bilateral TKA could not be conducted.

Asensio-Pascaul et al. [5] reported that baseline data did not differ between the groups, which aligns with the findings of this current study. According to their study, a well-functioning unilateral THA does not influence the functional outcomes of a subsequent ipsilateral TKA [5]. This was also seen in the results of this study; the functional outcomes did not differ between the TKA patients and the THA/TKA patients at the one- and five-year follow-ups. Liu et al. [9] also concluded that outcomes TKA in patients with a prior ipsilateral THA can be performed safely with excellent outcomes.

According to a study by Singh and Lewallen [10], the presence of ipsilateral joint involvement after two and five years of THA or TKA was strongly associated with poor pain and functional outcomes. They believe that the potential mechanisms involved include biomechanics, greater weight bearing on the THA/TKA, and limited ability to perform adequate rehabilitation and strengthening due to concomitant ipsilateral joint involvement.

In this study, THA/ TKA patients had slightly more pain at baseline than TKA patients. However, this difference was not statistically significant, and after the one- and five-year follow-ups, pain levels were similar in both groups. Lee et al. [6] found no statistical difference in pain between the TKA and THA/TKA patient groups during follow-ups after a minimum of four years.

LLD after arthroplasty can influence patients’ walking distances and lifestyles. It can alter the kinematics of the hip and lead to worse functional outcomes [5]. Data about the possible LLD of the patients was not available. Thus, investigating LLD´s influence on walking or physical function could not be done in this study.

Jungmann et al. [4] investigated the association of prevalent unilateral THA with degenerative changes in the ipsilateral and contralateral knee. They concluded that prevalent unilateral THA increases the risk of developing OA in the contralateral knee. If OA occurs in the other joints of the lower extremities, it can increase pain and decrease walking distance. According to Singh and Lewallen [10], OA or other forms of arthritis in multiple joints, a failing primary or revision arthroplasty in the ipsilateral joint, and diseases of the periarticular structures, which lead to articular and periarticular symptoms, can lead to the involvement of the ipsilateral knee/hip in patients with primary THA and TKA [11, 12].

The knee can initially be very painful and swollen after TKA. Sometimes the healing and rehabilitation process after TKA is difficult. Recovery after primary THA is quite straightforward and generally better than recovery process after primary TKA. When the patient has a new implant in the hip, the pain will decrease significantly or stop completely, and functional ability recovers faster than it does after TKA. Biomechanical changes and load redistribution resulting from previous THA may affect the ipsilateral knee [11, 12].

After arthroplasty, patients may walk better due the exercise and reduced fear of movement over time. Increasing walking distance requires practice. However, it is challenging to determine whether shorter walking distances and pain at follow-up are due to TKA symptoms or complications from previous THA.

In the current study the revision rates were low in both groups and did not differ between groups. Although there was no statistical difference, it was clinically significant. Detailed reasons for revisions were unavailable from the registry-based data. In the population-based study by Marsh et al. [13] 3.3% of patients had a revision TKA within 5 years of their primary TKA. However, they did not investigate TKA revision rate if the patient had earlier THA. In Bottle et al. [14] found a three-year revision rate of 2.2% for patients who had TKA. Liu et al. [9] retrospectively identified patients who underwent TKA followed by ipsilateral THA (THA-TKA) for rheumatoid arthritis or osteoarthritis in their case-control study. They reported a TKA survival rate of 4.3% at 8 years [9], which aligns with current study´s finding of a 5% survival rate.

The limitations of the current study include its small size. Another potential limitation is the information reported by patients, such as pain and walking distance. These details may not be accurate, as Vaughn et al. [15] reported. The registry-based study has also limitations.

Specific information for all patients may not be available. During the long follow-up period, several surgeons performed knee and hip arthroplasties. Although the operation techniques have remained almost the same, the implant models have changed over time.

Although the registry-based data was from only one hospital, the revision data was received from Finnish National Institute of Health and Welfare. The strength of this study is that the data were systematically collected based on the Hungerford score [7]. An orthopaedic surgeon objectively recorded the mobility, stability, deformity, contracture, and strength of the knee, the sub-items of the Hungerford score. In a study by Bach et al. [16], two independent observers studied four different scores and their interobserver correlations. They concluded that the interobserver correlation (> 0.8) for knee range of motion, flexion contracture, and extension of the knee was greater when using the Hungerford score.

## Conclusions

Based on this study, the walking distance improved more quickly in TKA patients than in THA/TKA patients. In both groups, the total score increased per surgery year, but no statistical differences were found between the groups. Patients who had undergone more than one arthroplasty in their lower extremities managed their lives and activities with regard to pain almost as well as those who had undergone only one knee arthroplasty.

## Data Availability

No datasets were generated or analysed during the current study.

## Abbreviations

CI: Confidence interval
IQR: Interquartile range
LLD: Leg length discrepancy
OA: Osteoarthritis
THA: Total hip arthroplasty
TKA: Total knee arthroplasty
